# Sex hormone-binding globulin regulation of androgen bioactivity in vivo: validation of the free hormone hypothesis

**DOI:** 10.1038/srep35539

**Published:** 2016-10-17

**Authors:** Michaël R. Laurent, Geoffrey L. Hammond, Marco Blokland, Ferran Jardí, Leen Antonio, Vanessa Dubois, Rougin Khalil, Saskia S. Sterk, Evelien Gielen, Brigitte Decallonne, Geert Carmeliet, Jean-Marc Kaufman, Tom Fiers, Ilpo T. Huhtaniemi, Dirk Vanderschueren, Frank Claessens

**Affiliations:** 1 Laboratory of Molecular Endocrinology, Department of Cellular and Molecular Medicine, KU Leuven, Herestraat 49 PO box 901, 3000 Leuven, Belgium; 2 Gerontology and Geriatrics, Department of Clinical and Experimental Medicine, KU Leuven, Herestraat 49 PO box 7003, Leuven, Belgium; 3 Department of Cellular and Physiological Sciences, University of British Columbia, 2350 Health Sciences Mall, V6T 1Z3 Vancouver, B.C., Canada; 4 RIKILT, European Union Reference Laboratory for Residues, Wageningen UR, Akkermaalsbos 2, 6708WB Wageningen, The Netherlands; 5 Clinical and Experimental Endocrinology, Department of Clinical and Experimental Medicine, KU Leuven, Herestraat 49 PO box 902, 3000 Leuven, Belgium; 6 INSERM UMR1011, University of Lille and Institut Pasteur de Lille, Lille, France; 7 Laboratory for Hormonology and Department of Endocrinology, Ghent University Hospital, De Pintelaan 185, 9000 Ghent, Belgium; 8 Institute of Reproductive and Developmental Biology, Department of Surgery and Cancer, Imperial College London, Hammersmith Campus, Du Cane Rd, London, W12 0NN, United Kingdom

## Abstract

Sex hormone-binding globulin (SHBG) is the high-affinity binding protein for androgens and estrogens. According to the free hormone hypothesis, SHBG modulates the bioactivity of sex steroids by limiting their diffusion into target tissues. Still, the *in vivo* physiological role of circulating SHBG remains unclear, especially since mice and rats lack circulating SHBG post-natally. To test the free hormone hypothesis *in vivo*, we examined total and free sex steroid concentrations and bioactivity on target organs in mice expressing a human *SHBG* transgene. SHBG increased total androgen and estrogen concentrations via hypothalamic-pituitary feedback regulation and prolonged ligand half-life. Despite markedly raised total sex steroid concentrations, free testosterone was unaffected while sex steroid bioactivity on male and female reproductive organs was attenuated. This occurred via a ligand-dependent, genotype-independent mechanism according to *in vitro* seminal vesicle organ cultures. These results provide compelling support for the determination of free or bioavailable sex steroid concentrations in medicine, and clarify important comparative differences between translational mouse models and human endocrinology.

A central dogma in endocrinology, the so-called “free hormone hypothesis”, states that the biological activity of hormones is determined by their free (i.e. non-protein-bound) concentrations^1^. In the case of androgens and estrogens, free hormone concentrations and bioactivity are believed to be determined by sex hormone-binding globulin (SHBG)^2^. SHBG is a liver-secreted homodimeric glycoprotein with high affinity (nM *K*_*d*_) for dihydrotestosterone (DHT), testosterone (T) and, to somewhat lesser extent, 17β-estradiol (E2)^3^. Androgens and estrogens are key regulators of reproductive organs as well as other sexually dimorphic tissues like muscle, adipose tissue and bone^4^. T can directly, or indirectly following conversion into DHT, act via the androgen receptor (AR), or via estrogen receptors (ERα and β) following aromatization of T into E2. In normal adults about 55% of T in men or E2 in women is bound to circulating SHBG. The remainder is loosely bound to bulk carrier proteins like albumin and only 1–3% of sex steroids normally circulate freely^5^. In clinical practice, free sex steroid concentrations are calculated from total sex steroid, albumin and SHBG concentrations, or measured directly using equilibrium dialysis or other methods^6^,^7^.

Despite widespread adoption of the free hormone hypothesis, it remains unclear whether total or free sex steroids are the most clinically useful measurement^5^,^8^. More importantly, experimental validation of the free hormone hypothesis in sex steroid biology remains scanty. In addition to the general belief that SHBG suppresses sex steroid bioactivity, several additional effects of SHBG have been proposed. Foremost among these are ligand-independent effects, actions of liganded SHBG via a membrane receptor or endocytosis, paradoxical prevention of sex steroid deficiency due to increased circulating ligand half-life (and thus availability), and regulation of the androgen/estrogen ratio^9^,^10^,^11^. Others have argued that higher levels of SHBG are compensated *in vivo* by hypothalamic-pituitary feedback, resulting in higher total sex steroid concentrations^12^. This results in great confusion and continuing debate over whether —and by which mechanisms— SHBG regulates total, free and/or bioavailable sex steroid concentrations and their physiological responses^11^,^12^,^13^,^14^.

These unanswered questions bear clinical relevance because SHBG concentrations fluctuate across the lifespan, are influenced by gender or drugs and have been associated with diseases including type 2 diabetes and metabolic syndrome, osteoporosis, reproductive disorders etc.^15^,^16^,^17^. Notably, several epidemiological studies have even suggested effects of SHBG independent from total or free sex hormone concentrations^16^,^18^. However, since SHBG concentrations are a sensitive indicator of metabolic status^19^,^20^, debate persists as to whether decreased SHBG and/or low T levels are a biomarker rather than a cause of disease^21^,^22^,^23^.

An SHBG homologue known as the androgen-binding protein is expressed locally in the testis of mice and rats but hepatic *Shbg* expression and secretion is lacking postnatally in these rodent models. Therefore a mouse model expressing a human SHBG transgene (SHBG-Tg) has been established previously^24^. Hitherto it has been used almost exclusively to study regulation of human *SHBG* expression *in vivo*^19^,^20^,^25^. Conversely, lack of circulating SHBG was found to explain why mice have remarkably low and highly fluctuating total T serum concentrations^4^,^24^, but otherwise no reproductive or other phenotypic effect of SHBG has yet been ascertained.

There is increasing awareness that reliable sex steroid measurements require mass spectrometry rather than immunoassays, not only in humans but also in rodent models^26^,^27^,^28^. This is especially the case for E2, the serum level of which has even been reported to be undetectable in male mice in recent mass spectrometry-based studies^26^,^28^. This virtual absence of circulating E2 in male mice has been used previously to criticize the ubiquitous use of male mice as models to study the effects of gender or sex steroids on chronic diseases like diabetes, osteoporosis etc.^4^. In the present study, we used the SHBG-Tg mouse model in combination with liquid chromatography tandem mass spectrometry (LC-MS/MS) methods to determine how, and by which mechanisms, SHBG influences circulating total and free sex steroid concentrations and their physiological effects on various target tissues (reproductive organs, muscle and bone mass, and glucose metabolism).

## Results

### SHBG increases circulating total concentrations of androgens, estrogens and their precursors

**Figure 1:**
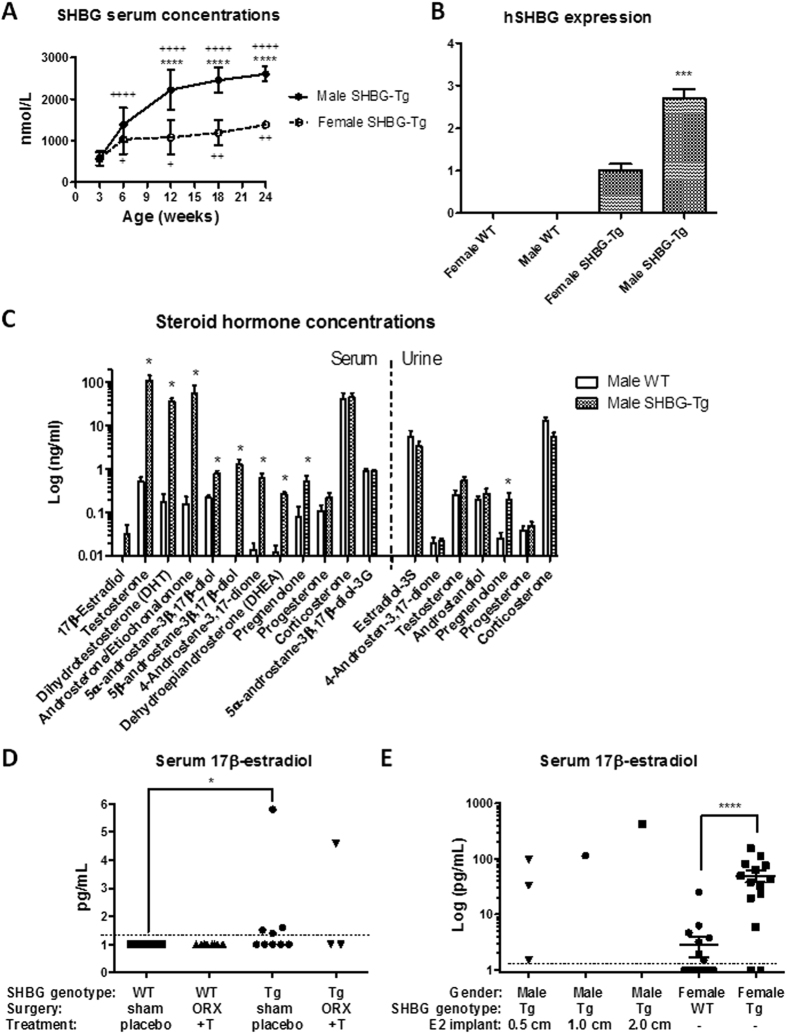
Endocrine profile of SHBG-Tg mice. (**A**) Human SHBG serum concentrations (shown as mean ± SD) in male and female SHBG-Tg mice of different ages (n = 7–10 per gender and age except 24-week-old females, n = 3). ****P < 0.0001 vs. female SHBG-Tg mice of same age group. ^+^P < 0.05, ^++^P < 0.01, ^++++^P < 0.0001 vs. 3-week-old mice of same gender. (**B**) Human SHBG expression (relative to mouse 18S) in livers of female and male WT and SHBG-Tg mice (n = 3 for WT and n = 5 for SHBG-Tg mice of each gender). ***P = 0.0002 for difference between SHBG-Tg females and males. (**C**) Concentrations in serum (left from vertical dotted line) and urine (right from vertical dotted line) of selected steroid hormones in 24-week-old WT and SHBG-Tg male mice (n = 5 per group). *P < 0.05 vs. WT mice. (**D**) Serum E2 in 12-week-old WT and SHBG-Tg male mice, either sham-operated and given empty placebo implants, or orchidectomized with continuous-release s.c. T replacement. The limit of quantification (LOQ) of the LC-MS/MS method (1.3 pg/mL) is indicated by the horizontal dotted line. *P < 0.05, n as indicated by individual replicates in each group. (**E**) Serum E2 in 12-week-old orchidectomized male mice given s.c. undiluted E2 implants of different lengths as indicated, or female WT and SHBG-Tg mice. The limit of quantification (LOQ) of the LC-MS/MS method (1.3 pg/mL) is indicated by the horizontal dotted line. ****P < 0.0001, n as indicated by individual replicates in each group.

SHBG serum concentrations in SHBG-Tg mice are shown in Fig. 1A (in WT mice, SHBG was undetectable, <1 nmol/L). SHBG levels increased with age in each gender but more so in males, and the sex difference was significant at 12 weeks of age. Hepatic expression of human *SHBG* was also higher in male than female SHBG-Tg mice (Fig. 1B). At 24 weeks of age, steroid hormone profiling of serum and urine by LC-MS/MS revealed that male SHBG-Tg mice have increased total serum concentrations of T and DHT (~200-fold), as well as several of their precursors (or metabolites) like pregnenolone, dehydroepiandrosterone (DHEA), androstenedione, androsterone/etiocholanolone and 5α- or 5β-androstane-3β,17β-diol (Fig. 1C). WT male mice (n = 5) had undetectable E2 concentrations whereas in SHBG-Tg males (n = 5), two had detectable circulating E2. In contrast, urinary concentrations of T or androstenedione, as well as conjugation products like estradiol-3-sulphate and 5α-androstane-3β, 17β-diol-3-glucuronide were unaffected. Progesterone or corticosterone levels in serum or urine were not different between WT and SHBG-Tg littermates. A similar profile was observed in 24-week-old female mice (unselected for estrous cycle phase) (Fig. S1A). Using a more sensitive, dedicated LC-MS/MS method^29^, E2 was undetectable in WT male mice, whereas in SHBG-Tg males (n = 9), values above the limit of quantification (1.3 pg/mL) were observed in four of them (Fig. 1D). These low to undetectable values could not be attributed to the known fluctuating T concentrations in male mice^30^, as shown by the consistent lack of E2 in WT mice with a s.c. continuous-release T implant (Fig. 1D). The lack of E2 was also not due to limitations of the LC-MS/MS method, as shown in castrated SHBG-Tg males with s.c. silicone E2 implants, or female mice (Fig. 1E). E2 concentrations measured randomly in the estrous cycle were increased in female SHBG-Tg vs. WT mice (Fig. 1E). These differences are summarized schematically along with a diagram of murine steroid metabolism in Fig. S1B.

### Male SHBG-Tg mice display a mild hypogonadal phenotype

**Figure 2:**
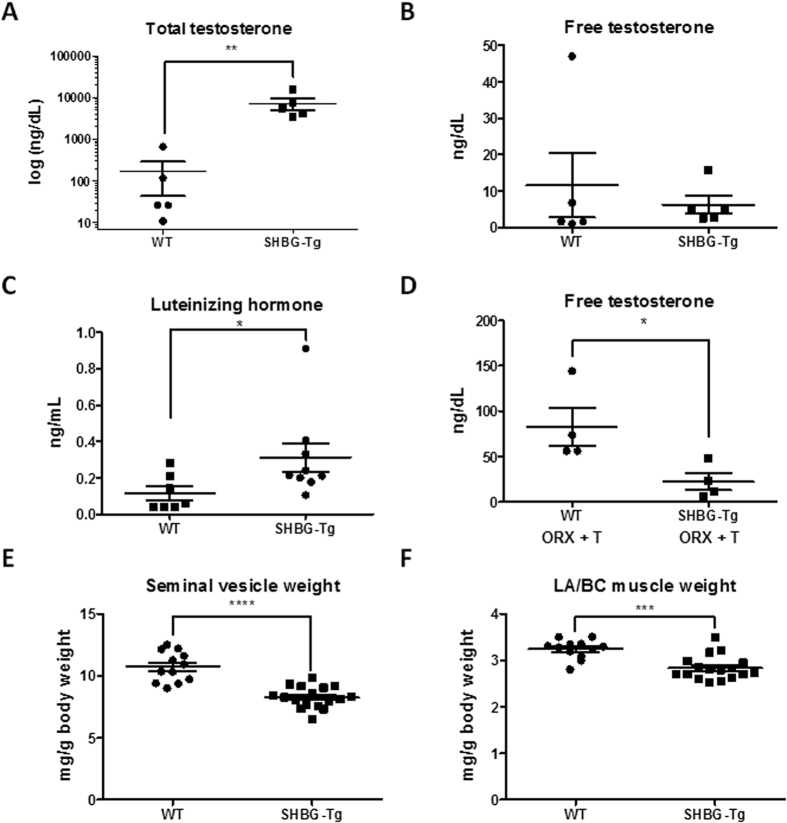
Evidence of hypogonadism in 24-week-old male SHBG-Tg mice. (**A,B**) Serum total and free T. n = 5 biological replicates per group (to obtain sufficient volume, sera of 2 mice were pooled if necessary). **P < 0.01 (**C**) Serum luteinizing hormone (n = 7–9 per group). *P < 0.05. (**D**) Free T by equilibrium dialysis in WT and SHBG-Tg mice which were orchidectomized and given s.c. T replacement (ORX+T). n = 4 per group, *P < 0.05. (**E,F**) Seminal vesicle and levator ani/bulbocavernosus complex (LA/BC) muscle weights. Individual replicates, mean ± SEM are shown. ***P < 0.001, ****P < 0.0001.

Despite the higher total T concentrations in SHBG-Tg mice, their free T concentrations measured directly by equilibrium dialysis coupled to LC-MS/MS were unaltered (Fig. 2A,B). However, serum luteinizing hormone (LH) concentrations were increased in SHBG-Tg male mice (Fig. 2C). To confirm that the latter hypothalamic-pituitary feedback regulation normalized the free T concentrations, SHBG-Tg mice were castrated and given T implants. Under these conditions, free T levels were decreased in SHBG-Tg males (Fig. 2D). The weight of the most androgen-sensitive organs (seminal vesicles (SV) and levator ani/bulbocavernosus [LA/BC] muscles) was decreased at 24 weeks of age (Fig. 2E,F). Body weight was not different between SHBG-Tg and WT mice at any age (Fig. S2A). The decrease in SV and LA/BC muscle weights was evident as early as 9 weeks of age (late puberty) (Fig. S2B,D). The difference in SV weight was entirely attributable to lower SV fluid content (Fig. S2C). The anogenital distance, which is greater in male compared to female mice due to androgen effects, was not influenced by SHBG at 3, 6, 9 or 12 weeks of age (Fig. S2E). Lean body mass, fat mass or bone mineral density by DXA, as well as the response to an i.p. glucose or insulin tolerance test at 24 weeks, showed gender differences as expected, which however did not differ by SHBG genotype (Fig. S3A–F).

**Figure 3:**
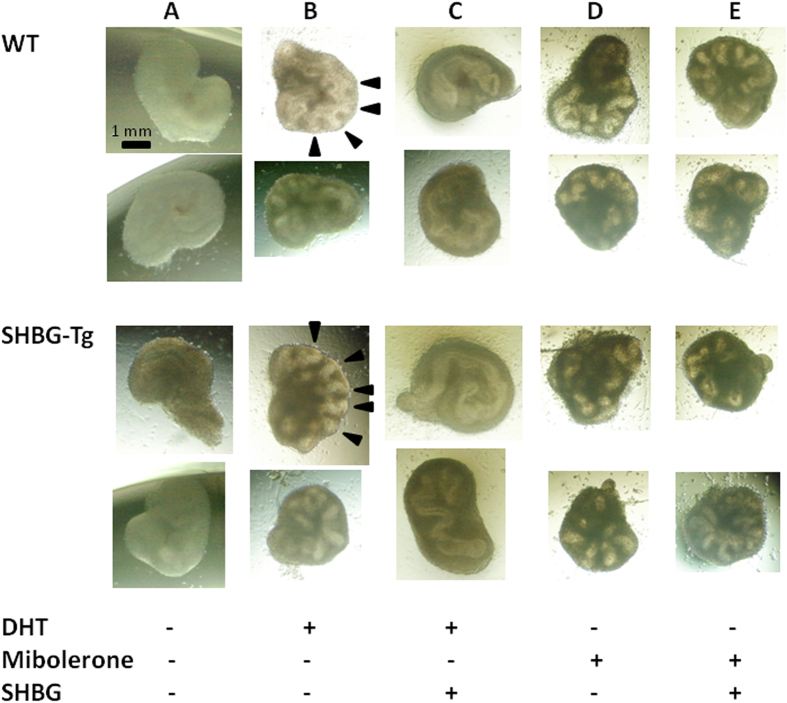
Effect of SHBG on androgen-induced branching morphogenesis in seminal vesicle organ cultures. For each genotype, two representative microphotographs at day 3 of culture are displayed. All pictures scaled identically (1 mm scale bar shown in first photograph only). (**A**) Unstimulated condition showing lack of epithelial folding in the absence of androgens. (**B**) Induction of branching morphogenesis (arrowheads in two panels) by 1 nM DHT in both genotypes. (**C**) Suppression of DHT-induced branching morphogenesis by SHBG in the media. (**D**) Induction of epithelial folding by 1 nM mibolerone in both genotypes. (**E**) Lack of suppression of mibolerone-induced branching morphogenesis by SHBG.

To investigate whether the reduced SV weight was due to an intrinsic, genotype-dependent developmental deficit or an endocrine mechanism, we performed organ cultures. SVs dissected from day 0–1 postnatal mice showed no morphologic differences at baseline between WT and SHBG-Tg mice (data not shown). After three days, either 1nM of DHT or mibolerone (a potent synthetic AR agonist) stimulated branching morphogenesis, regardless of donor mouse genotype (Fig. 3A,B,D). Compared to control medium, SHBG-containing medium suppressed the induction of epithelial folding by DHT (Fig. 3C), but not by mibolerone (which has negligible affinity for SHBG) (Fig. 3E).

### SHBG protects against male and female reproductive organ hypertrophy

**Figure 4:**
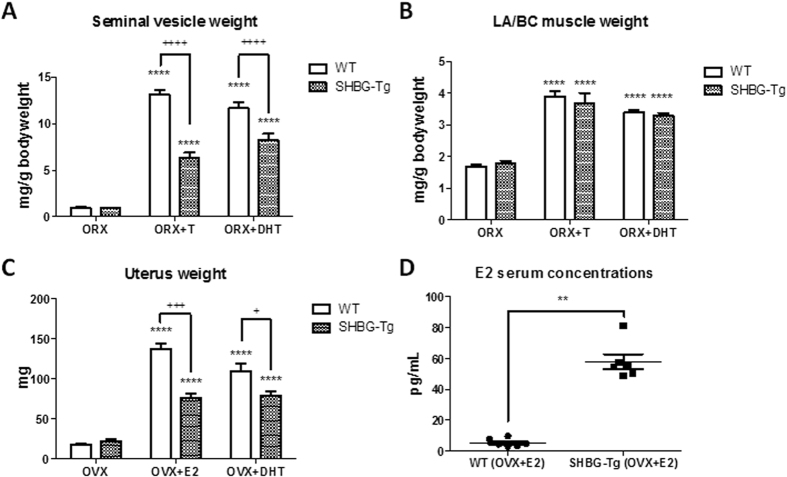
SHBG inhibits the stimulatory effects of sex steroids on male and female reproductive organs. (**A,B**) Seminal vesicle and LA/BC muscle weight of 12-week-old WT and SHBG-Tg mice following 3 weeks of orchidectomy (ORX), with placebo, testosterone (ORX+T) or DHT replacement (ORX+DHT). n = 5–10 for each genotype and treatment group. ****P < 0.0001 compared to ORX in same genotype. ^++++^P < 0.0001 vs. WT in same treatment group. (**C**) Uterus weight of female WT or SHBG-Tg mice following ovariectomy (OVX) with either placebo, E2 (diluted 1/16 w/w with cholesterol) replacement (OVX+E2) or DHT replacement (OVX+DHT) replacement between 18 and 20 weeks of age, respectively. n = 5–14 per group. (**D**) Serum E2 concentrations in WT and SHBG-Tg following OVX+E2 replacement (n = 6 randomly selected mice per group). **P  < 0.01 vs. WT by Mann-Whitney U test.

Next, we investigated the response of SHBG-Tg and WT mice to castration and androgen replacement. In castrated mice with s.c. T or DHT implants, SV hypertrophy was prevented in SHBG-Tg compared to WT males (Fig. 4A). The hypertrophic response of LA/BC muscles was however unaffected (Fig. 4B). There were no differences in the weight of SVs or LA/BC muscle in castrated SHBG-Tg vs. WT mice. In ovariectomized mice, uterus weights were not different between WT and SHBG-Tg females. However, the effect of both E2 and DHT^31^ on uterus weight was less pronounced in SHBG-Tg females (Fig. 4C), despite the fact that total E2 concentrations were *higher* in OVX+E2-treated SHBG-Tg than WT female mice (Fig. 4D). Under basal conditions however, randomly cycling 24-week-old female mice displayed no difference in uterus weight (Fig. S2F).

### SHBG increases the half-life of circulating ligands and prevents their entry into target tissues

**Figure 5:**
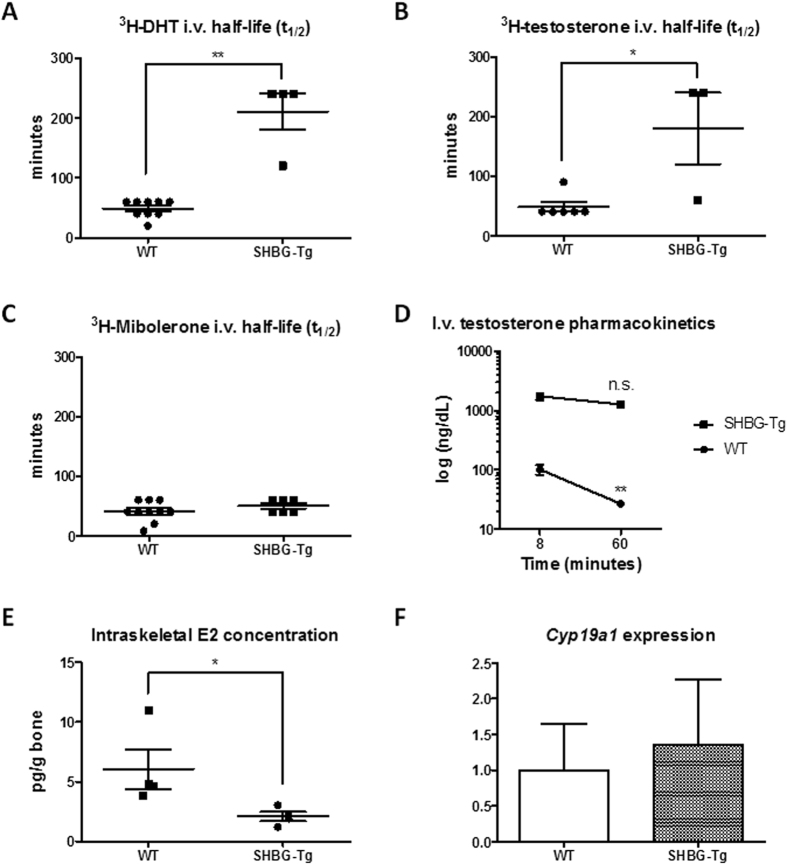
Effects of SHBG on sex steroid pharmacokinetics and biodistribution. (**A**) Half-life of tritium-labeled DHT following i.v. injection. n = 4–9 per group. **P < 0.01. (**B**) Half-life of tritium-labeled T following i.v. injection. n = 3–6 per group. *P < 0.05. (**C**) Half-life of tritium-labeled mibolerone following i.v. injection. n = 6–10 per group. (**D**) Testosterone concentrations at 8 or 60 minutes following i.v. T injection. n = 6–10 per group. **P < 0.01 compared to 8 minute timepoint in same genotype. (**E**) Concentration of E2 in bone homogenates. n = 4 per group. *P < 0.05. (**F**) Relative mRNA expression of mouse aromatase (*Cyp19a1*) in the bones (tibia and femur) of 24-week-old WT and SHBG-Tg male mice. n = 9–11 per group.

A single i.v. injection of tritium-labeled DHT, T and mibolerone into castrated mice revealed that SHBG prolonged the half-life of the tracer signal following DHT and T but not mibolerone injection (Fig. 5A–C). To further exclude the possibility of confounding by tracer catabolism, the experiment was repeated using unlabeled T. Total T peak concentrations measured by LC-MS/MS were ~20-fold increased eight minutes after i.v. injection of SHBG-Tg mice. After 60 minutes, T in WT mice was 74% lower compared to the eight minute time point, whereas SHBG-Tg mice showed a non-significant 28% T decrease (Fig. 5D).

To reconcile the undetectable circulating E2 in WT and very low circulating E2 concentrations in SHBG-Tg male mice with the well known physiological effects of estrogens in male mice and the presence of estradiol-3-sulphate in urine, we investigated local E2 concentrations within a classical target tissue like bone. Homogenates from appendicular bone of mice with s.c. T implants (see Fig. 4A,B, ORX+T group) revealed clear intraskeletal concentrations of E2 in all mice, despite undetectable circulating E2 in all these animals. Furthermore, intraskeletal E2 was lower in SHBG-Tg than in WT males (Fig. 5E), while *Cyp19a1* expression was unaffected (Fig. 5F). However, no clear bone phenotype could be discerned under basal conditions in 24-week-old mice (Fig. S4A–F).

## Discussion

Our findings provide for the first time clear experimental validation of the free hormone hypothesis with respect to sex steroids, i.e. that SHBG-bound T in the circulation is restricted from entering into target tissues and eliciting its physiological functions *in vivo*. Unexpectedly however, the most striking effect of SHBG is not to decrease free T concentrations, but to increase total androgen and estrogen concentrations (Fig. 1). This occurs via at least two mechanisms: hypothalamic-pituitary feedback stimulation (as evidenced by increased LH and concentrations of precursor androgens which themselves do not bind to SHBG) and increased circulating ligand half-life (Fig. 5A–D). This feedback regulation normalized free T levels in response to differences in circulating SHBG, resulting in only minimal residual suppression of androgen bioactivity evident only in the most sensitive organs (SVs and LA/BC muscles) under basal conditions.

The conclusion that SHBG increases total but decreases bioactive sex steroid concentrations is in line with human genetic evidence. A genome-wide association meta-analysis identified a functional SHBG polymorphism (rs6258) which decreased total and increased free T levels in adult men^13^. One man with undetectable SHBG also had very low total but normal free T levels^32^. Although measured free T was not significantly different in SHBG-Tg male mice (Fig. 2B), this represents only one time point which could have been confounded by the known highly fluctuating, spiking T concentrations in male mice^30^. The somewhat wider distribution of free T in WT mice and the difference in free T in mice given a continuous-release s.c. T implant (Fig. 2D) supports this possibility.

In humans, protection from endogenous or exogenous androgen excess is thought to be a key physiological function of SHBG. For example, low SHBG may reinforce the hirsutism of obese women with polycystic ovary syndrome^33^ while frank virilization from fetal androgens has been observed in a woman with genetic SHBG deficiency^34^,^35^. A similar role in protecting against hormone excess has been demonstrated for other binding proteins^36^, and our findings in orchidectomized and ovariectomized mice treated with T or DHT support this possibility.

Interestingly, SHBG prevented SV hypertrophy but did not interfere with the hypertrophic response of the androgen-sensitive LA/BC muscles (Fig. 4B), which resembles the effect of a selective androgen-receptor modulator (SARM)^37^. Because T treatment in older men may adversely affect reproductive tissues like the prostate, alternative treatment strategies are needed. The free hormone hypothesis acknowledges that the effect of binding proteins may differ between tissues depending on their vascularity, blood flow rate or lipid composition, for example^1^,^15^. Besides differences in tissue characteristics, a possible explanation for this SARM-like effect is that SHBG has the highest affinity for DHT, which is a crucial mediator of androgen effects on reproductive but not other organs^38^. However, further investigation is needed to confirm whether SHBG truly has tissue-specific effects, and whether SHBG concentrations also modulate the response to T therapy in humans.

In any case, a trade-off of this protection against hyperstimulation was mild hypogonadism under basal conditions, which was evident only in the most sensitive target organs (SV and LA/BC muscle weights, Fig. 2E,F). This is consistent with our recent finding that men with high SHBG but normal total T may still have symptoms and signs consistent with late-onset male hypogonadism^39^. Importantly however, the high SHBG concentrations in SHBG-Tg mice had no effects on less androgen-sensitive outcomes like body composition, bone or glucose metabolism, suggesting that the numerous epidemiological studies suggesting this possibility^15^,^16^,^18^ may suffer from residual confounding by metabolic status^19^,^40^,^41^. These findings are in line with another recent study showing that obesity-induced metabolic derangements regulate hepatic SHBG expression, whereas SHBG itself did not prevent the metabolic consequences of obesity^41^. We also found no phenotypic evidence for ligand-independent effects of SHBG, since no differences were seen in gonadectomized or pre-pubertal animals.

**Figure 6:**
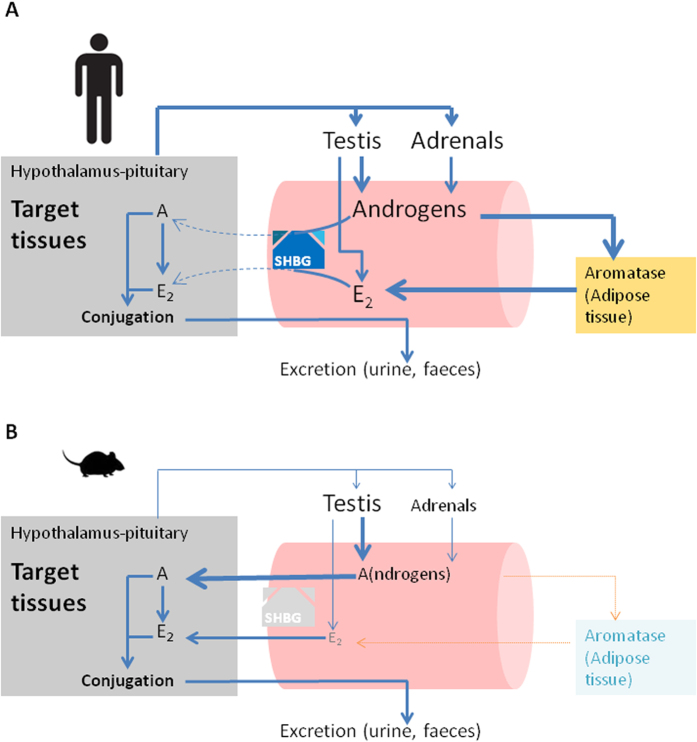
Summary of differences in sex steroid endocrinology between men and mice. (**A**) In men, circulating androgens are derived from the testes and adrenals. Circulating E2 is estimated to be ~85% derived from aromatization in peripheral adipose tissues, with the remainder secreted directly from the testis. SHBG binds androgens as well as estrogens and prevents their entry into target tissues (indicated by striped arrows). Within target tissues, T can also be converted locally into E2. Conjugation and feedback regulation occur within tissues and thus at the level of the free/bioactive sex steroids. Water-soluble conjugation products are removed by hepatic and renal clearance. (**B**) In contrast, male mice have lower androgen and undetectable E2 concentrations due to (a) less pronounced adrenal secretion of precursor steroids, (b) possibly due to markedly lower peripheral aromatization due to absence in mice of the alternative promoter which drives peripheral CYP19A1 expression in humans, and (c) lack of SHBG, which facilitates rapid entry of both sex steroids into target tissues. Thus, estrogens act mainly as local hormones in target tissues of male mice. Lower gonadotropin concentrations in male mice are at least in part due to lack of SHBG.

Finally, several important lessons for laboratory animal sciences can be drawn from our results. First, our findings reinforce the notion that reproductive organ weights are more reliable indicators of sex steroid status than circulating sex steroid concentrations in mice and rats (species which lack circulating SHBG)^4^. In other words, total T levels are lower in male mice compared to humans not because lab mice are hypogonadal^30^, but because of lack of circulating SHBG. The results in SHBG-Tg mice also unambiguously demonstrate the presence of circulating precursor androgens like DHEA, despite lower adrenal sex steroid production in rodents compared to humans^42^,^43^. Further misinterpretation of rodent endocrinology derives from reliance on immunoassays^30^ rather than mass spectrometry to measure very low concentrated analytes^27^,^28^. Thirdly, we show that even in SHBG-Tg male mice circulating E2 is very low (compared to humans or female mice). These findings should however be interpreted with caution because even in SHBG-Tg mice, the serum E2 concentrations were either below or only slightly above the limit of quantification (LOQ) of our method. Nevertheless, these results support the conclusions that lack of detectable circulating E2 in male mice is not due to fluctuating T concentrations and only partially explained by lack of SHBG. Still, estrogen conjugation products were clearly present in urine and E2 has well established physiological effects in male target organs like bone^4^. We reasoned that, contrary to the situation in humans where circulating E2 derives predominantly from aromatization in peripheral adipose tissues^44^, E2 in mice probably derives mainly from local aromatization within target tissues (Fig. 6). Indeed, mice are known to have low aromatase expression in adipose tissue^45^. Our hypothesis was supported by demonstrating higher intraskeletal than circulating E2 in castrated, T-treated mice of both genotypes, with lower concentrations in SHBG-Tg compared to WT animals (Fig. 5E). The difference in intraskeletal E2 concentrations between genotypes could not be attributed to altered aromatase (*Cyp19a1*) gene expression (Fig. 5F) and is therefore likely explained by the fact that SHBG restricts the bioavailability of T. However, it should be noted that aromatase expression was very low in bones of both WT and SHBG-Tg mice, in line with previous studies^46^. Further work is therefore needed to examine whether intraskeletal E2 truly derives from local aromatization within bone, or from an endocrine mechanism whereby it is aromatized in other nearby or distant tissues with subsequent rapid uptake into bone and clearance from the serum due to lack of SHBG. In any case, given that E2 replacement almost inevitably produces supraphysiological E2 concentrations in male mice (Fig. 1C), we caution against deriving physiologically relevant conclusions from such an approach. Finally, it should be noted that serum SHBG concentrations are higher in SHBG-Tg mice compared to humans^25^. Moreover, we observed an age-related increase in both genders but more so in male SHBG-Tg mice, similar to previous findings^25^. In humans however, men have higher, lower and similar SHBG concentrations compared to women before puberty, after puberty and in old age, respectively^15^. Explaining the age- and gender-specific differences in SHBG concentrations and transgene expression in SHBG-Tg mice was not the aim of our study, but previous studies found little evidence that sex steroids directly regulate hepatic SHBG expression and secretion in SHBG-Tg mice^25^. Thus, investigation into the indirect effects of age and gender via other e.g. metabolism-related transcription factors may be required^20^,^47^.

In summary, our detailed examination of the SHBG-Tg model reveals that contrary to the general misconception that SHBG decreases free T concentrations, *in vivo* it mainly increases total androgen and estrogen concentrations (via hypothalamic-pituitary feedback and prolonged circulating half-life). Nevertheless, SHBG attenuated androgen bioactivity resulting in mild hypogonadal signs in reproductive organs, but no major phenotypic effects on other sexually dimorphic target tissues. These findings offer empirical support for measuring free or bioavailable T in clinical practice, since SHBG can clearly confound interpretations based on circulating total sex steroid concentrations. Although we found no evidence that SHBG has ligand-independent effects *in vivo*, alternative mechanisms of action beyond the regulation of sex steroid plasma transport may still contribute to effects on certain organs^9^,^10^. Finally, our study may assist in the appropriate use of male mouse models for translational biomedical research.

## Methods

### SHBG-Tg mice

Heterozygous SHBG_(4.3kb)_^+/−^ mice (SHBG-Tg)^24^ and WT littermate controls were housed in conventional facilities. Presence of the transgene was confirmed by PCR on tail DNA using previously described primer pairs^47^. Mice were backcrossed for eight generations to obtain an incipient congenic (>99.5%) C57BL/6J background confirmed by a Jax 1500 SNP panel (n = 2 mice). The KU Leuven animal ethics committee approved all procedures (P028/2012). Animals were bred and all experimental methods were performed in accordance with the Belgian national regulations for Animal Welfare and the 2010/63/EU directive. For *ex vivo* analyses, animals were euthanized by cardiac puncture following pentobarbital anesthesia. Tissues were dissected and wet weight measured. SVs were weighed before and after manual expression of SV fluid.

### Surgical procedures

Mice were anesthetized by i.p. ketamin-xylazine (100/25 μg/kg) or isoflurane anesthesia and given 60 μg/kg buprenorphine analgesia. Orchidectomy and ovariectomy were performed via suprapubic and bilateral flank incisions. Implants of medical-grade silicone tubing (Silclear, Degania Medical, Degania, Israel) sealed with medical adhesive silicone (Silastic, Biesterfeld, Germany) were implanted in the nuchal region, either empty (placebo) or filled with T, DHT or various doses of E2 (all from Sigma-Aldrich, St. Louis, MO, USA). Mice were operated at 9 weeks of age and *ex vivo* analyses were performed at 12 weeks of age unless noted otherwise.

### Serum analyses

Cardiac or tail blood was collected in Eppendorf or Microvette serum tubes (Sarstedt, Nümbrecht, Germany) respectively, left to clot at room temperature for >1 h and centrifuged twice at 10,000 g for 5 minutes according to manufacturer instructions. Human SHBG concentrations were measured on a Roche Modular E clinical immunoassay platform. Mouse LH was measured at Türkü Center for Disease Modeling with a supersensitive immunofluorometric assay as described^48^. Osteocalcin was measured by an in-house radioimmunoassay^49^.

### Mass spectrometry and equilibrium dialysis

Total T and E2 were measured at the University Hospitals Leuven by LC-MS/MS without derivatization using a two-dimensional chromatography system and an AB/Sciex QTrap 5500 tandem mass spectrometer in atmospheric pressure chemical ionization positive (APCI) and electrospray ionization (ESI) negative ion mode, respectively, as described^29^ (and Antonio *et al*., *manuscript in prep.*).

Endogenous steroid hormone profiling was performed at Waegeningen RU by an UPLC-MS/MS method in analogy with a previous reported method^50^ (and Blokland *et al*., *manuscript in preparation*). Solid phase extraction of aglycons and sulphate/glucuronide conjugates was achieved using Oasis HLB and WAX 96-well plates, respectively, followed by derivatization of aglycons with picolinic acid. Aglycons and conjugates were chromatographically separated on BEH and CSH C^18^ columns respectively, and analyzed separately on a Xevo TQ-S mass spectrometer (Waters Corp., Milford, MA, USA) in ESI mode switching between positive and negative ion selection mode.

Total T as well as free T after equilibrium dialysis were measured at the University Hospital of Ghent by LC-MS/MS using an AB/Sciex QTrap 5500 tandem mass spectrometer in APCI mode as described^51^. Briefly, free T was directly measured by LC-MS/MS in the dialysate, after overnight equilibrium dialysis of 500 μL serum at 37 °C using Fast Micro-Equilibrium dialyzer cartridges with regenerated cellulose 25 kD membranes (Harvard Apparatus; Holliston, USA). Total interassay CV for direct measurement of free T by equilibrium dialysis coupled to LC-MS/MS is 8%.

### Neonatal seminal vesicle cultures

Neonatal SV cultures were performed according to^52^ with some modifications (G. Cunha, *personal communication*). Left and right SVs were dissected from postnatal day 0–1 pups and incubated for 3 days at 37 °C, 5% CO_2_ and 100% humidity in 96-well plates (Greiner bio-one, Frickenhausen, Germany) with 50 μL of DMEM/F12 media with insulin (10 mg/L), transferrin (5.5 mg/L) and selenium (6.7 ng/L) (ThermoFisher Scientific (Waltham, MA, USA). DHT, mibolerone (Toronto Research Chemicals, Ontario, Canada) or vehicle were added in 1:1000 dilutions with or without 60 nmol/L SHBG from conditioned media obtained by transfecting HEK293-cells with a human SHBG or empty pRC/CMV vector^3^. Transfected cell media were then concentrated by centrifugal ultrafiltration using Amicon Ultracel tubes with a 30 K molecular weight cut-off (Merck, Darmstadt, Germany).

### *In vivo* pharmacokinetics

Tritium-labeled DHT, T and mibolerone (Perkin-Elmer, Waltham, MA, USA) or unlabeled T (Sigma-Aldrich, St. Louis, MO, USA) were added to 50 μL of autologous serum of each mouse, incubated for >30 minutes and injected via the lateral tail vein. Tailbleeding was performed at several timepoints, for each of which 10 μL of serum was dissolved in 3 mL of UltimaGold scintillation cocktail and analyzed on a Tri-Carb 2810 TR liquid scintillation counter (Perkin-Elmer, Waltham, MA, USA). Half-life was estimated as the first timepoint at which the tracer signal was >50% below peak values in each animal. Unlabeled T was analyzed in serum obtained from cardiac puncture by in-house LC-MS/MS as described above.

### Quantitative PCR

Total RNA was extracted from livers as well as left tibias and femurs of 24-week-old mice using TRIzol reagent (Invitrogen), according to the manufacturer’s protocols. After digestion with DNase I (Qiagen, Antwerp, Belgium), cDNA was synthesized from 1 μg RNA using the RevertAid M-MuLV Reverse Transcriptase kit (Fermentas, St Leon-Rot, Germany) and random hexamer primers (Fermentas). The PCR reaction mixtures (10 μl) contained 1× Fast SYBR Green qPCR Master mix (ThermoFisher Scientific) and 0.15 μL (2.5 μM) of each primer. The StepOne Plus Real-Time PCR system (Applied Biosystems, Foster City, CA, USA) was used. The primers used were: human SHBG-Fw 5′-ATCACAAAAACCTCCTCCTCCTT-3′. SHBG-Rev 5′-ATCTCCCATCATCCAGCCGT-3′ (190 Bp amplicon), mouse *Cyp19a1*-Fw 5′-TGGCAAGCTCTCCTCATCAA-3′, *Cyp19a1*-Rev 5′-TCTCCACGTCTCTCAGCGA-3′ (200 Bp amplicon), mouse *18S*-Fw 5′-CGCCGCTAGAGGTGAAATTC-3′, *18S*-Rev 5′-TTGGCAAATGCTTTCGCTC-3′. All primers were designed using NCBI Primer-BLAST software to hybridize to different exons and generate a single amplicon in melting curve assays. Gene expression was quantified using the relative (ΔΔCT) method with normalization to the levels of 18S ribosomal RNA.

### Other *in vivo* procedures

Mice were weighed using electronic scales. Anogenital distance was measured by an investigator blinded to genotype using a 0.1 mm Vernier caliper (Scienceware,Wayne, NJ, USA) with the median value of 5 measurements recorded. Glucose and insulin tolerance testing was performed after 16 or 6 h fasting and i.p. injection of 2.5 mg glucose or 0.75 mU of insulin/g body weight, respectively. Glycemia was measured using Accu-Check Aviva glucostrips. Whole body (minus head) lean, fat and bone mass was determined by *in vivo* dual energy X-ray absorptiometry using the PIXImus mouse densitometer (Lunar Corp) with an ultrahigh resolution (0.18 × 0.18 pixels, 1.6 line pairs/mm) and software v1.45.

### Bone microCT and homogenization

Femurs and L5 vertebra were scanned using a Skyscan 1172 *ex vivo* microCT (Brüker, Kontich, Belgium) with 50 kV, 200 μA, 5 μm resolution, 0.5 mm aluminum filter and 0.6° angular rotation step settings, and analyzed using NRecon software as described previously^53^.

For intraskeletal E2 measurements, bilateral femurs, tibias and humeri were dissected, cleaned of soft tissue and snap frozen in liquid N2. Pools of 200–300 mg were homogenized using CKMix50-R tubes on a Precellys 24 homogenizer (Bertin Technologies, Montigny le Bretonneux, France). Methanol, water and internal standards were added and LC-MS/MS performed as described above for serum samples.

### Statistical analyses

Differences in continuous measures between two or more groups were analyzed by unpaired t-test (or Mann-Whitney U-test if variances were unequal) or ANOVA with Dunett’s post-test, respectively. Two-way ANOVA with Bonferroni post-test was used to examine genotype-by-treatment interactions. Mean and standard error (SEM) are shown in all graphs unless specified otherwise. Analyses were performed using Graphpad Prism v5.04, and two-tailed P < 0.05 was considered significant.

## Additional Information

**How to cite this article**: Laurent, M. R. *et al*. Sex hormone-binding globulin regulation of androgen bioactivity in vivo: validation of the free hormone hypothesis. *Sci. Rep.* **6**, 35539; doi: 10.1038/srep35539 (2016).

## Supplementary Material

Supplementary Information

## References

[b1] Mendel C. M. The free hormone hypothesis: a physiologically based mathematical model. Endocr Rev 10, 232–274 (1989). 2673754 10.1210/edrv-10-3-232

[b2] Bhasin S. . Reference ranges for testosterone in men generated using liquid chromatography tandem mass spectrometry in a community-based sample of healthy nonobese young men in the Framingham Heart Study and applied to three geographically distinct cohorts. J Clin Endocrinol Metab 96, 2430–2439 (2011). 21697255 10.1210/jc.2010-3012 PMC3146796

[b3] Wu T. S. & Hammond G. L. Naturally occurring mutants inform SHBG structure and function. Mol Endocrinol 28, 1026–1038 (2014). 24892637 10.1210/me.2014-1058 PMC5414826

[b4] Vanderschueren D. . Sex steroid actions in male bone. Endocr Rev 35, 906–960 (2014). 25202834 10.1210/er.2014-1024 PMC4234776

[b5] Rosner W., Auchus R. J., Azziz R., Sluss P. M. & Raff H. Position statement: Utility, limitations, and pitfalls in measuring testosterone: an Endocrine Society position statement. J Clin Endocrinol Metab 92, 405–413 (2007). 17090633 10.1210/jc.2006-1864

[b6] Zakharov M. N. . A multi-step, dynamic allosteric model of testosterone’s binding to sex hormone binding globulin. Mol Cell Endocrinol 399, 190–200 (2015). 25240469 10.1016/j.mce.2014.09.001

[b7] Vermeulen A., Verdonck L. & Kaufman J. M. A critical evaluation of simple methods for the estimation of free testosterone in serum. J Clin Endocrinol Metab 84, 3666–3672 (1999). 10523012 10.1210/jcem.84.10.6079

[b8] Basaria S. Testosterone levels for evaluation of androgen deficiency. JAMA 313, 1749–1750 (2015). 25942727 10.1001/jama.2015.4179

[b9] Rosner W., Hryb D. J., Kahn S. M., Nakhla A. M. & Romas N. A. Interactions of sex hormone-binding globulin with target cells. Mol Cell Endocrinol 316, 79–85 (2010). 19698759 10.1016/j.mce.2009.08.009

[b10] Hammes A. . Role of endocytosis in cellular uptake of sex steroids. Cell 122, 751–762 (2005). 16143106 10.1016/j.cell.2005.06.032

[b11] Khosla S. Editorial: Sex hormone binding globulin: inhibitor or facilitator (or both) of sex steroid action? J Clin Endocrinol Metab 91, 4764–4766 (2006). 17148570 10.1210/jc.2006-1990

[b12] de Ronde W. . Serum levels of sex hormone-binding globulin (SHBG) are not associated with lower levels of non-SHBG-bound testosterone in male newborns and healthy adult men. Clin Endocrinol (Oxf) 62, 498–503 (2005). 15807883 10.1111/j.1365-2265.2005.02252.x

[b13] Ohlsson C. . Genetic determinants of serum testosterone concentrations in men. PLoS Genet 7, e1002313 (2011). 21998597 10.1371/journal.pgen.1002313 PMC3188559

[b14] Laurent M. R. & Vanderschueren D. Reproductive endocrinology: functional effects of sex hormone-binding globulin variants. Nat Rev Endocrinol 10, 516–517 (2014). 25048036 10.1038/nrendo.2014.120

[b15] Hammond G. L., Wu T. S. & Simard M. Evolving utility of sex hormone-binding globulin measurements in clinical medicine. Curr Opin Endocrinol Diabetes Obes 19, 183–189 (2012). 22531107 10.1097/MED.0b013e328353732f

[b16] Eriksson A. L. . SHBG gene promoter polymorphisms in men are associated with serum sex hormone-binding globulin, androgen and androgen metabolite levels, and hip bone mineral density. J Clin Endocrinol Metab 91, 5029–5037 (2006). 16926255 10.1210/jc.2006-0679

[b17] Caldwell J. D. & Jirikowski G. F. Sex hormone binding globulin and aging. Horm Metab Res 41, 173–182 (2009). 18956301 10.1055/s-0028-1093351

[b18] Vandenput L. . High Serum SHBG Predicts Incident Vertebral Fractures in Elderly Men. J Bone Miner Res 31, 683–689 (2016). 26391196 10.1002/jbmr.2718 PMC4832265

[b19] Selva D. M., Hogeveen K. N., Innis S. M. & Hammond G. L. Monosaccharide-induced lipogenesis regulates the human hepatic sex hormone-binding globulin gene. J Clin Invest 117, 3979–3987 (2007). 17992261 10.1172/JCI32249 PMC2066187

[b20] Simó R., Sáez-López C., Barbosa-Desongles A., Hernández C. & Selva D. M. Novel insights in SHBG regulation and clinical implications. Trends Endocrinol Metab 26, 376–383 (2015). 26044465 10.1016/j.tem.2015.05.001

[b21] Hsu B. . Associations between circulating reproductive hormones and SHBG and prevalent and incident metabolic syndrome in community-dwelling older men: the Concord Health and Ageing in Men Project. J Clin Endocrinol Metab 99, E2686–E2691 (2014). 25259909 10.1210/jc.2014-2464

[b22] Hsu B. . Reproductive Hormones and Longitudinal Change in Bone Mineral Density and Incident Fracture Risk in Older Men: The Concord Health and Aging in Men Project. J Bone Miner Res 30, 1701–1708 (2015). 25736139 10.1002/jbmr.2493

[b23] Antonio L. . Associations between sex steroids and the development of metabolic syndrome: a longitudinal study in European men. J Clin Endocrinol Metab 100, 1396–1404 (2015). 25636052 10.1210/jc.2014-4184

[b24] Jänne M., Deol H. K., Power S. G., Yee S. P. & Hammond G. L. Human sex hormone-binding globulin gene expression in transgenic mice. Mol Endocrinol 12, 123–136 (1998). 9440816 10.1210/mend.12.1.0050

[b25] Jänne M., Hogeveen K. N., Deol H. K. & Hammond G. L. Expression and regulation of human sex hormone-binding globulin transgenes in mice during development. Endocrinology 140, 4166–4174 (1999). 10465289 10.1210/endo.140.9.7004

[b26] McNamara K. M. . Measurement of sex steroids in murine blood and reproductive tissues by liquid chromatography-tandem mass spectrometry. J Steroid Biochem Mol Biol 121, 611–618 (2010). 20144714 10.1016/j.jsbmb.2010.02.001

[b27] Handelsman D. J. . Measurement of testosterone by immunoassays and mass spectrometry in mouse serum, testicular, and ovarian extracts. Endocrinology 156, 400–405 (2015). 25365769 10.1210/en.2014-1664

[b28] Nilsson M. E. . Measurement of a Comprehensive Sex Steroid Profile in Rodent Serum by High-Sensitive Gas Chromatography-Tandem Mass Spectrometry. Endocrinology 156, 2492–2502 (2015). 25856427 10.1210/en.2014-1890

[b29] Pauwels S. . Sensitive routine liquid chromatography-tandem mass spectrometry method for serum estradiol and estrone without derivatization. Anal Bioanal Chem 405, 8569–8577 (2013). 23892882 10.1007/s00216-013-7259-5

[b30] Michiel Sedelaar J. P., Dalrymple S. S. & Isaacs J. T. Of mice and men–warning: intact versus castrated adult male mice as xenograft hosts are equivalent to hypogonadal versus abiraterone treated aging human males, respectively. Prostate 73, 1316–1325 (2013). 23775398 10.1002/pros.22677 PMC4009979

[b31] Simitsidellis I., Gibson D. A., Cousins F. L., Esnal-Zufiaurre A. & Saunders P. T. A Role for Androgens in Epithelial Proliferation and Formation of Glands in the Mouse Uterus. Endocrinology 157, 2116–2128 (2016). 26963473 10.1210/en.2015-2032 PMC4870887

[b32] Vos M. J., Mijnhout G. S., Rondeel J. M., Baron W. & Groeneveld P. H. Sex hormone binding globulin deficiency due to a homozygous missense mutation. J Clin Endocrinol Metab 99, E1798–E1802 (2014). 24937543 10.1210/jc.2014-2055

[b33] Tosi F. . Implications of Androgen Assay Accuracy in the Phenotyping of Women With Polycystic Ovary Syndrome. J Clin Endocrinol Metab 101, 610–618 (2016). 26695861 10.1210/jc.2015-2807

[b34] Hogeveen K. N. . Human sex hormone-binding globulin variants associated with hyperandrogenism and ovarian dysfunction. J Clin Invest 109, 973–981 (2002). 11927624 10.1172/JCI14060 PMC150924

[b35] Hammond G. L. Diverse roles for sex hormone-binding globulin in reproduction. Biol Reprod 85, 431–441 (2011). 21613632 10.1095/biolreprod.111.092593 PMC4480437

[b36] Safadi F. F. . Osteopathy and resistance to vitamin D toxicity in mice null for vitamin D binding protein. J Clin Invest 103, 239–251 (1999). 9916136 10.1172/JCI5244 PMC407885

[b37] Dubois V. . Enobosarm (GTx-024) Modulates Adult Skeletal Muscle Mass Independently of the Androgen Receptor in the Satellite Cell Lineage. Endocrinology 156, 4522–4533 (2015). 26393303 10.1210/en.2015-1479

[b38] Bhasin S. . Effect of testosterone supplementation with and without a dual 5alpha-reductase inhibitor on fat-free mass in men with suppressed testosterone production: a randomized controlled trial. JAMA 307, 931–939 (2012). 22396515 10.1001/jama.2012.227 PMC6035750

[b39] Antonio L. . Low Free Testosterone is Associated with Hypogonadal Signs and Symptoms in Men with Normal Total Testosterone. J Clin Endocrinol Metab 101, 2647–2657 (2016). 26909800 10.1210/jc.2015-4106

[b40] Selva D. M. & Hammond G. L. Peroxisome-proliferator receptor gamma represses hepatic sex hormone-binding globulin expression. Endocrinology 150, 2183–2189 (2009). 19179433 10.1210/en.2008-1289

[b41] Saez-Lopez C., Rivera-Gimenez M., Hernandez C., Simo R. & Selva D. M. SHBG-C57BL/ksJ-db/db: A New Mouse Model to Study SHBG Expression and Regulation During Obesity Development. Endocrinology 156, 4571–4581 (2015). 26441241 10.1210/en.2015-1677

[b42] Callewaert F. . Sexual dimorphism in cortical bone size and strength but not density is determined by independent and time-specific actions of sex steroids and IGF-1: evidence from pubertal mouse models. J Bone Miner Res 25, 617–626 (2010). 19888832 10.1359/jbmr.090828

[b43] van Weerden W. M., Bierings H. G., van Steenbrugge G. J., de Jong F. H. & Schroder F. H. Adrenal glands of mouse and rat do not synthesize androgens. Life Sci 50, 857–861 (1992). 1312193 10.1016/0024-3205(92)90204-3

[b44] Labrie F. Intracrinology. Mol Cell Endocrinol 78, C113–C118 (1991). 1838082 10.1016/0303-7207(91)90116-a

[b45] Zhao H. . A humanized pattern of aromatase expression is associated with mammary hyperplasia in mice. Endocrinology 153, 2701–2713 (2012). 22508516 10.1210/en.2011-1761 PMC3359608

[b46] Sjögren K. . Elevated aromatase expression in osteoblasts leads to increased bone mass without systemic adverse effects. J Bone Miner Res 24, 1263–1270 (2009). 19257817 10.1359/jbmr.090208

[b47] Selva D. M., Hogeveen K. N. & Hammond G. L. Repression of the human sex hormone-binding globulin gene in Sertoli cells by upstream stimulatory transcription factors. J Biol Chem 280, 4462–4468 (2005). 15574421 10.1074/jbc.M409616200

[b48] Haavisto A. M. . A supersensitive immunofluorometric assay for rat luteinizing hormone. Endocrinology 132, 1687–1691 (1993). 8462469 10.1210/endo.132.4.8462469

[b49] Verhaeghe J., Van Herck E., Van Bree R., Van Assche F. A. & Bouillon R. Osteocalcin during the reproductive cycle in normal and diabetic rats. J Endocrinol 120, 143–151 (1989). 2783957 10.1677/joe.0.1200143

[b50] Blokland M. H., Van Tricht E. F., Van Rossum H. J., Sterk S. S. & Nielen M. W. Endogenous steroid profiling by gas chromatography-tandem mass spectrometry and multivariate statistics for the detection of natural hormone abuse in cattle. Food Addit Contam Part A Chem Anal Control Expo Risk Assess 29, 1030–1045 (2012). 22651818 10.1080/19440049.2012.675593

[b51] Fiers T. . A critical evaluation of salivary testosterone as a method for the assessment of serum testosterone. Steroids 86, 5–9 (2014). 24793565 10.1016/j.steroids.2014.04.013

[b52] Shima H., Tsuji M., Young P. & Cunha G. R. Postnatal growth of mouse seminal vesicle is dependent on 5 alpha-dihydrotestosterone. Endocrinology 127, 3222–3233 (1990). 2249647 10.1210/endo-127-6-3222

[b53] Callewaert F. . Differential regulation of bone and body composition in male mice with combined inactivation of androgen and estrogen receptor-alpha. FASEB J 23, 232–240 (2009). 18809737 10.1096/fj.08-113456

