# Supplementary material for: Sex hormone-binding globulin regulation of androgen bioactivity in vivo: validation of the free hormone hypothesis
Source: Sci Rep. 2016 Oct 17;6:35539. doi: 10.1038/srep35539 (PMC5066276; doi:10.1038/srep35539)
Supplement: Supplementary Information [file srep35539-s1.pdf]

Sex hormone-binding globulin regulation of androgen bioactivity in vivo: validation of the free hormone hypothesis

Michaël R. Laurent,[1,2] Geoffrey L. Hammond,[3] Marco Blokland,[4] Ferran Jardí,[5] Leen Antonio,[5] Vanessa Dubois,[1,6] Rougin Khalil,[5] Saskia S. Sterk,[4] Evelien Gielen,[2] Brigitte Decallonne,[5] Geert Carmeliet,[5] Jean-Marc Kaufman,[7] Tom Fiers,[7] Ilpo T. Huhtaniemi,[8] Dirk Vanderschueren,[5] and Frank Claessens[1*]

[1] Laboratory of Molecular Endocrinology, Department of Cellular and Molecular Medicine, KU Leuven, Herestraat 49 PO box 901, 3000 Leuven, Belgium

[2] Gerontology and Geriatrics, Department of Clinical and Experimental Medicine, KU Leuven, Herestraat 49 PO box 7003, Leuven, Belgium

[3] Department of Cellular and Physiological Sciences, University of British Columbia, 2350 Health Sciences Mall, V6T 1Z3 Vancouver, B.C., Canada

[4] RIKILT, European Union Reference Laboratory for Residues, Wageningen UR, Akkermaalsbos 2, 6708WB Wageningen, The Netherlands

[5] Clinical and Experimental Endocrinology, Department of Clinical and Experimental Medicine, KU Leuven, Herestraat 49 PO box 902, 3000 Leuven, Belgium

[6] INSERM UMR1011, University of Lille and Institut Pasteur de Lille, Lille, France

[7] Laboratory for Hormonology and Department of Endocrinology, Ghent University Hospital, De Pintelaan 185, 9000 Ghent, Belgium

[8] Institute of Reproductive and Developmental Biology, Department of Surgery and Cancer, Imperial College London, Hammersmith Campus, Du Cane Rd, London, W12 0NN, United Kingdom

Supplementary information

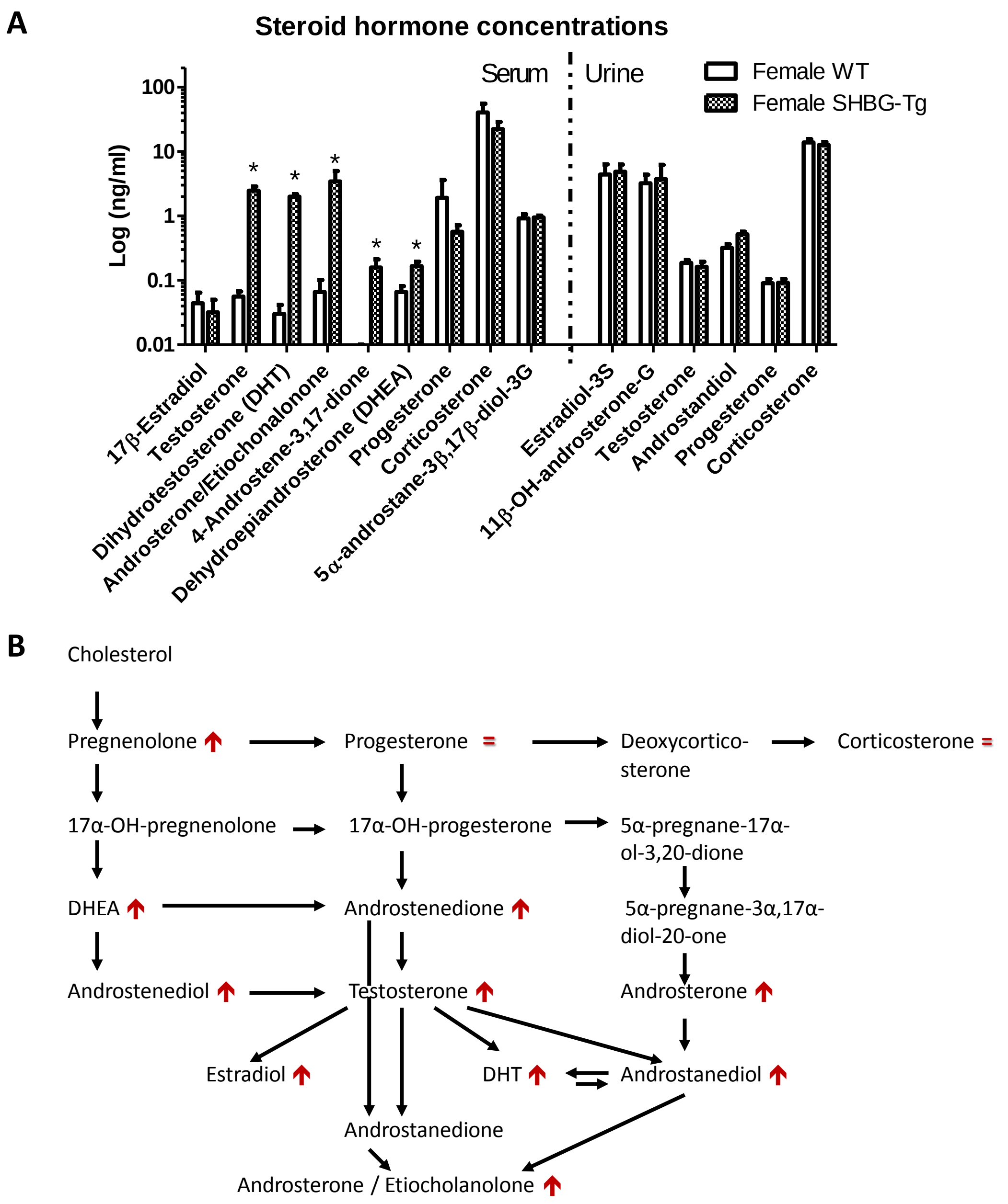

Fig. S1. Steroid hormone profile in SHBG-Tg mice.

A. Concentrations in serum (left from vertical dotted line) and urine (right from vertical dotted line) of selected steroid hormones in randomly cycling 24-week-old WT and SHBG-Tg female mice (n=5 per group). * = P<0.05 vs. WT mice.

B. Simplified diagram showing the metabolism of selected steroid hormones (assessed by our steroid profile LC-MS/MS method) along with summary of differences (shown in red) in SHBG-Tg vs. WT mice.

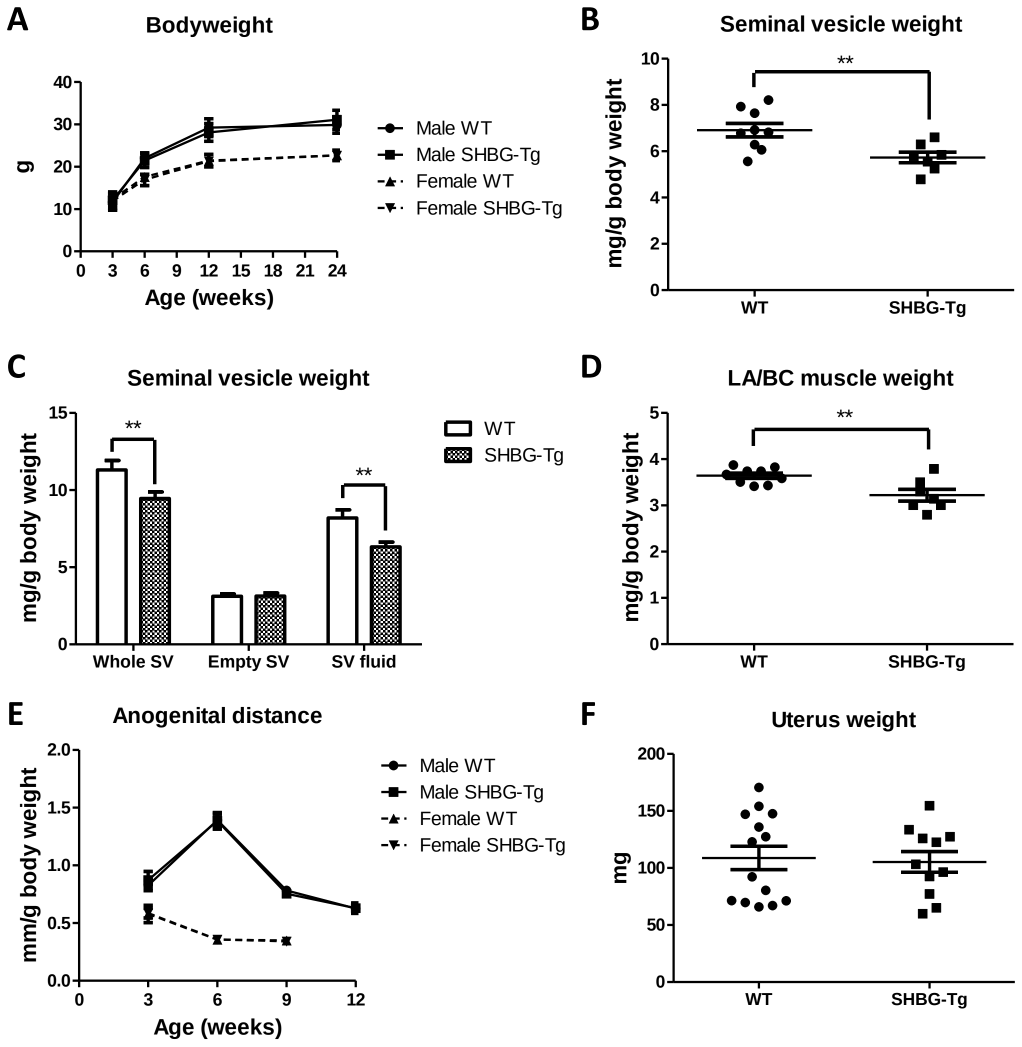

Fig. S2. Additional phenotyping of SHBG-Tg mice.

A. Body mass evolution in both genders and genotypes at different ages. n=8-17 per group

B. Seminal vesicle (SV) weights at 9 weeks of age. n=7-10 per group. ** = P<0.01

C. Weights of intact SVs, fluid-emptied organs and SV fluid. n=4-6 per group. ** = P<0.01 by Bonferroni's post-test after two-way ANOVA showing significant effect of fluid expression (P<0.0001), genotype (P=0.03) and interaction (P=0.001).

D. Levator ani/bulbocavernosus complex (LA/BC) muscle weights at 9 weeks of age. n=7-10 per group. ** = P<0.01

E. Anogenital distance evolution in both genders and genotypes at different ages across puberty. Note consistently higher levels in males, and decrease in males from age 6 weeks due to faster body weight gains than anogenital separation. Mean ± SD shown, n=5-13 per group and timepoint.

F. Uterus weights of WT and SHBG-Tg mice at 24 weeks of age. n=11-14 per group.

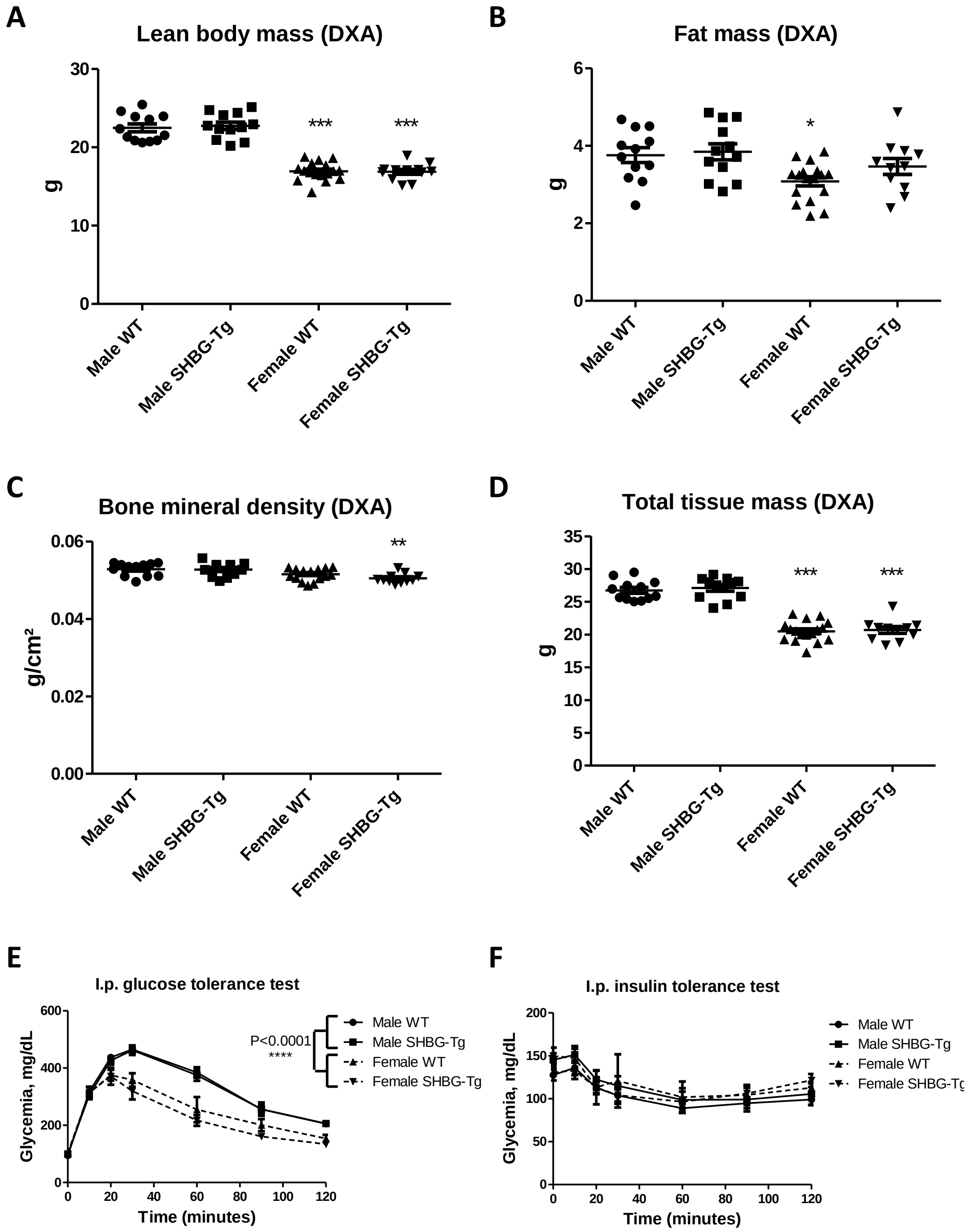

Fig. S3. Normal body composition and glucose homeostasis in SHBG-Tg mice.

A-D. Lean body mass, fat mass, bone mineral density and total tissue mass by *in vivo* dual-energy X-ray absorptiometry (DXA) in 24-week-old male and female WT and SHBG-Tg mice. n=11-17 per group. * = P<0.05, ** = P<0.01, *** = P<0.001 compared to WT males.

E, F. Glycemic responses to an i.p. glucose or insulin tolerance test. n=7-8 (males) and n=4-6 (females) for each genotype.

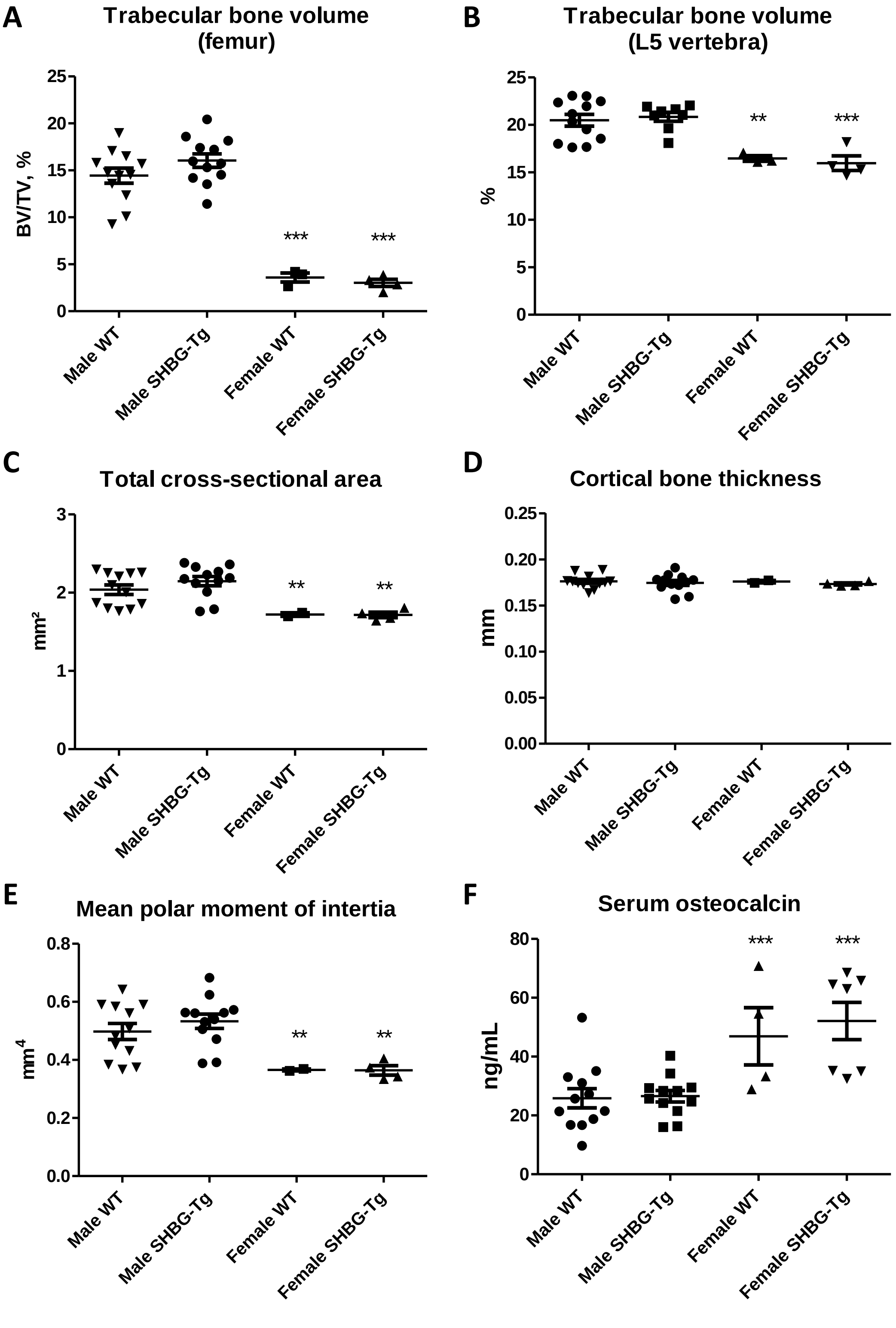

Fig. S4. Lack of bone phenotype in 24-week-old SHBG-Tg mice.

A. Trabecular bone volume (BV/TV) in the distal femur. n=12 (males) and n=3-4 (females) for each genotype. *** = P<0.001 compared to WT males.

B. Trabecular bone volume (BV/TV) in the L5 vertebra. n=8-12 (males) and n=3-4 (females) for each genotype. ** = P<0.01, *** = P<0.001 compared to WT males.

C. Total cross-sectional bone area (an indicator of periosteal circumference) of the femoral midshaft. n=12 (males) and n=3-4 (females) for each genotype. ** = P<0.01 compared to WT males.

D. Cortical bone thickness of the femoral midshaft. n=12 (males) and n=3-4 (females) for each genotype.

E. Mean polar moment of intertia of the femoral midshaft. n=12 (males) and n=3-4 (females) for each genotype. ** = P<0.01 compared to WT males.

F. Serum osteocalcin. n=12 (males) and n=4-7 (females) for each genotype.
